# Equine-Assisted Interventions (EAIs) for Children with Autism Spectrum Disorders (ASD): Behavioural and Physiological Indices of Stress in Domestic Horses (*Equus caballus*) during Riding Sessions

**DOI:** 10.3390/ani11061562

**Published:** 2021-05-27

**Authors:** Laura Contalbrigo, Marta Borgi, Marta De Santis, Barbara Collacchi, Adele Tuozzi, Marica Toson, Veronica Redaelli, Rosangela Odore, Cristina Vercelli, Annalisa Stefani, Fabio Luzi, Emanuela Valle, Francesca Cirulli

**Affiliations:** 1 Istituto Zooprofilattico Sperimentale delle Venezie, Viale Dell’Università, 10, 35020 Legnaro, Italy; mdesantis@izsvenezie.it (M.D.S.); mtoson@izsvenezie.it (M.T.); astefani@izsvenezie.it (A.S.); 2 Centre for Behavioural Sciences and Mental Health, Istituto Superiore di Sanità, Viale Regina Elena 299, 00161 Rome, Italy; marta.borgi@iss.it (M.B.); barbara.collacchi@iss.it (B.C.); adele.tuozzi@gmail.com (A.T.); francesca.cirulli@iss.it (F.C.); 3 Department of Biomedical, Surgical and Dental Sciences—One Health Unit, Via Pascal, 36, 20133 Milano, Italy; veronica.redaelli@unimi.it (V.R.); fabio.luzi@unimi.it (F.L.); 4 Department of Veterinary Sciences, University of Torino, Largo P. Braccini 2, 10095 Grugliasco, Italy; rosangela.odore@unito.it (R.O.); cristina.vercelli@unito.it (C.V.); emanuela.valle@unito.it (E.V.)

**Keywords:** equine-assisted interventions, horse, autism, infrared thermography, behaviour, heart rate, heart rate variability, welfare, cortisol, catecholamines

## Abstract

**Simple Summary:**

Equine-assisted interventions (EAIs) are gaining increasing attention as complementary practices in autism spectrum disorders (ASD) as they can provide physical and psychological enrichment to children. However, ASD children could manifest inappropriate behaviours, potentially affecting the welfare of horses. This multicentre study aimed to investigate behavioural and physiological indices of stress in horses involved in EAI standardised sessions with children with ASD compared to sessions involving typically developing (TD) children. It followed a controlled within-subject design with repeated measurements involving 19 horses and 38 children. We compared behavioural and physiological responses of horses between sessions and among session phases. Results indicate a lower sympathetic tone in horses involved in ASD sessions, while in the mounting and dismounting phases, the horses displayed behavioural signs of stress, independently from children’s behaviour. Results from this study indicate that professionals should increase their awareness of horse’s welfare and refine methodologies used in EAIs.

**Abstract:**

Equine-assisted interventions (EAIs) are well-known complementary practices combining physical activity with emotional/cognitive stimulation. They are especially suited for children with autism spectrum disorders (ASD) who need a high degree of physical and psychological enrichment. Even though EAIs have become a common practice, stress responses in horses interacting with individuals that can manifest inappropriate behaviours, such as ASD children, have not been thoroughly investigated. Our multicentre study aimed to investigate behavioural and physiological indices of stress in horses involved in EAI standardised sessions with children with ASD compared to typically developing (TD) children. A controlled within-subject design with repeated measurements involving 19 horses and 38 children was adopted. Stress-related behaviours, heart rate, heart rate variability, and eye temperature were recorded during the riding sessions. Moreover, blood samples were collected from horses before and after each session to monitor changes in blood adrenocorticotropic hormone (ACTH), cortisol, and catecholamines. Results indicate that, in general, stress responses in horses involved in EAIs did not differ as a function of the horse being ridden by children with ASD or TD. A lower sympathetic tone in horses involved in ASD sessions was found, while in the mounting and dismounting phases, horses displayed behavioural signs of stress, independently from children’s behaviour. We conclude that professionals working in EAI should increase their awareness of animal welfare and refine riding practices, taking into account horse’s needs.

## 1. Introduction

Equine-assisted interventions (EAIs) are gaining increasing attention as complementary practices for the rehabilitation of persons with mental and physical disabilities [1,2]. These interventions combine physical activity with emotional/cognitive elements and such a combination has made these programmes suitable for rehabilitation programmes targeting children in need of a high degree of stimulation, such as children with autism spectrum disorders (ASD) [3,4,5]. Positive effects of EAIs include improvements in areas of functioning known to be impaired in ASD. The most affected areas are social responsiveness and motivation, language/communication, stress behaviours (e.g., irritability and hyperactivity), motor functioning, and sensory processing [6,7,8,9,10,11,12]. Although the involvement of animals in ASD rehabilitation programmes has become a common practice (an estimated one in four children with ASD has participated in animal-assisted interventions) [13], the response of animals when interacting with subjects with social and emotional problems—like those diagnosed with ASD—have not been sufficiently investigated. There is preliminary evidence suggesting that horses are sensitive to riders’ behaviours, especially when the rider has emotional or behavioural problems [14]. Some studies show that their relational style and conflicting orders may cause distress to the horse [15,16] which is potentially associated with long-term detrimental health and behavioural effects [17]. As an example, inconsistent application of communicative signals can cause confusion and conflict-related behaviours in horses, with possible negative consequences for their welfare, as well as for human safety [18,19,20]. 

As far as we know, no studies have so far examined horses’ reaction to EAI sessions with children diagnosed with ASD, whose core symptoms affect their relational and communicative behaviour, often showing impulsivity, hyperactivity, and aggressiveness [21,22].

Within this framework, our study aimed to investigate behavioural and physiological indices of stress in horses involved in EAI sessions with children with ASD versus typically developing children to appraise which activities and interactions cause more discomfort in the animal. Stress-related behaviours during the sessions were recorded concurrently with measures of physiological functions such as heart rate, heart rate variability, and eye temperature, which are known to provide insight into responses of the autonomic nervous system (ANS) in a range of physiological and pathological conditions [23,24,25,26]. Moreover, changes in blood ACTH, cortisol, and catecholamines were assessed before and after each session. The use of non-invasive techniques, such as infrared thermography and heart rate monitoring, to collect physiological data during riding sessions has the advantage that it does not interfere with the spontaneous child-horse interaction and minimizes the stress caused by classic sampling procedures. 

## 2. Materials and Methods 

### 2.1. Ethical Statement

The methods and procedures of this study were in accordance with Italian legislation (D.L.vo n. 26 of 4 March 2014) on the protection of animals used for scientific purposes (Authorization n°1055/2015-PR). Owners of horses gave written consent to the enrolment of their animals in the study. Moreover, children’s parents signed informed written consent for the involvement of their children in riding sessions, and all data about their children were processed in compliance with privacy and data protection law. The research protocol was approved by the Ethics Committees of the Istituto Zooprofilattico Sperimentale delle Venezie (EC protocol number 6/2014) and the Istituto Superiore di Sanità (Prot. PRE-790/15).

### 2.2. Horses

Nineteen horses of different ages (mean 17.3 ± 5.7 years), sex (six mares and thirteen geldings), and breed (4 Italian saddles, 8 ponies, 3 Argentine, 1 Maremmano, 1 Haflinger, 1 Wielkopolski, and 1 Hungarian) were recruited from 4 Italian Riding Centres. We collected data about their long-term and recent anamnesis, training procedures, and weekly workload. All horses were suitable for morphology and biomechanics; they were in good health without signs of injuries, sickness, or disease and they didn’t show abnormal behaviours and stereotypes. They were involved in a 1 h EAI session and 2 h of riding school each day. All of them were trained for EAI using operant conditioning with positive reinforcement, without punishment and coercive methods. They had an average of 5 years of experience in EAIs. All horses were normally group-housed in the paddock during the day and they spent the night in an individual box. They have free access to water. The pasture was supplemented with hay and additional horse feed twice a day. Husbandry practices, veterinary care, and grooming of horses in the four riding centres were similar.

### 2.3. Human Subjects

Thirty-eight children, aged 6–12 years, were involved in the study: 19 with a previous ASD diagnosis and 19 typically developing (TD) children. Children were selected among those who attended the riding centres participating in the study. All children had a similar experience with horses: less than one year. Exclusion criteria were horse allergy and/or fear.

### 2.4. Study Design

This multicentre study followed a controlled within-subject design with repeated measurements. Each horse was involved in two different riding sessions:

1. An equine-assisted session with the horse ridden by a child with ASD (ASD session).
2. Control session with the horse ridden by a TD child (TD session).

Horses were randomly assigned to a child with ASD and a TD child using a predetermined randomised order generated in Microsoft Excel. Sessions were carried out on two consecutive days. The order of ASD session and TD session was counterbalanced among subjects. To reduce bias due to the environment and the circadian/circannual effects, all sessions were performed in the afternoon (between 2 pm and 4 pm) during the winter season (November 2015–January 2016).

### 2.5. Setting

ASD and TD sessions were carried out outside, in a quiet environment. Each riding centre had a fenced area assigned to EAIs and riding sessions with soft and well-drained soil. Sessions were carried out by professionals with good knowledge of each horse. In each riding centre, the same team was involved in all sessions. It was composed of a horse handler and a therapist who was in charge of the ASD child or the TD child during the whole session, according to Italian National Guidelines for Animal-assisted interventions [27]. Both ASD and TD sessions followed a standardised procedure (adapted from Borgi and colleagues [3]). They lasted about 30 min and followed the protocol described in Table 1. 

### 2.6. Experimental Procedure

**Figure 1:**
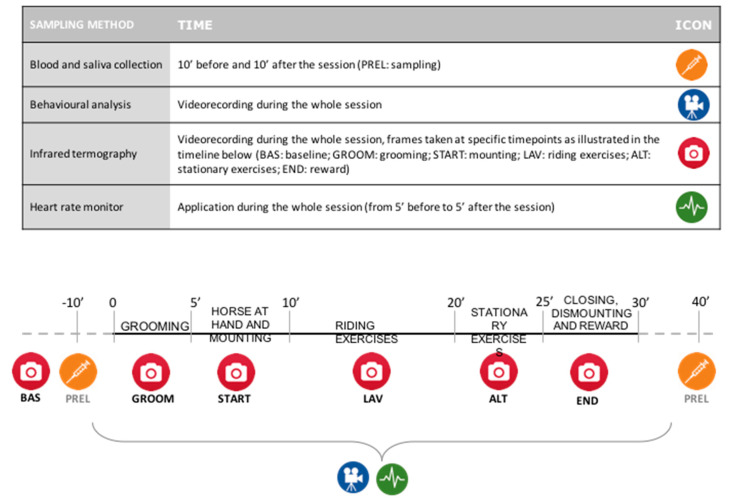
Sampling protocol adopted in the study.

The experimental procedure is plotted in Figure 1, which provides a detailed description of collection methods of physiological and behavioural data from the horses enrolled in the study. 

#### 2.6.1. Blood Sampling

Blood samples were collected using jugular venipuncture [28] ten minutes before (−10’ = T0) and ten minutes after the end of each session (40’ = T1) to measure serum cortisol, plasma ACTH, and catecholamines—adrenaline, noradrenaline, and dopamine. Blood was collected with the horse standing in his box. We used K3EDTA tubes to get plasma and tubes without anticoagulants for serum. We centrifuged the K3EDTA tubes immediately after the collection whereas blood without anticoagulant was allowed to clot for 30 min in a vertical position. After centrifugation, each plasma sample was shared in 4 aliquots and serum in one tube. All samples were stored at −20 °C immediately. Afterward, samples were sent to the laboratory with a shipment in dry ice. Serum cortisol and plasma ACTH were analysed using the Immulite® automated chemiluminescence immunoassay system already validated for horse cortisol [29,30] and ACTH [31,32]. Plasma catecholamines (adrenaline, noradrenaline, and dopamine) were analysed using the CatCombi ELISA kit (IBL International GmbH). This kit allows the use of plasma from many animal species, including horses, since catecholamines have the same chemical structure in all animals and the catecholamine assays include an extraction step at the beginning of the procedure [33].

#### 2.6.2. Behavioural Analysis

All 38 sessions (19 ASD sessions and 19 TD sessions) were video-recorded with two Sony cameras (Handycam DCR-SX21E, Sony Europe B.V.The Heights, Brooklands, Weybridge, Surrey, KT13 0XW, United Kingdom)). The ethogram was developed in order to identify stress-related or discomfort behaviours in horses, such as snorting, pawing, head-shaking, head-tossing, as well as redirected activities indicating a conflict in the animal, such as oral manipulation and nodding [15,34,35,36,37,38,39,40,41]. All behaviours considered are listed in Table 2. Behavioural analysis was performed using a focal sampling method [42] and dedicated software (BORIS, Università degli Studi di Torino, Turin, Italy) [43]. In order to improve the reliability of behavioural data, behaviours were scored independently by two raters in a sub-sample of videos (13 videos of 13 different horses). An intraclass correlation coefficient (ICC) was calculated to test agreement between raters. The correlation between the two scores was positive and significant (ICC, r = 0.8, *p* < 0.05). After having reached the agreement, only one rater scored all videos. Behaviours were calculated as the relative frequency in each phase of the session described in Table 1: (i) grooming; (ii) horse at hand; (iii) mounting; (iv) riding exercises; (v) stationary exercises; (vi) closing (vii) dismounting; (viii) reward.

#### 2.6.3. Infrared Thermography (IRT)

Thermographic images were collected using an infrared camera (Thermo GEAR G120EX, Nippon Avionics Co., LTD, Ikonobecho, Japan). This camera has a thermal sensitivity of 0.04 °C at 30 °C and an accuracy of ±2% between 0 °C and 40 °C. Temperature measurements range between −40 °C to 1500 °C. Thermal videos were analysed through the InfRec Analyzer NS9500 Lite, a thermal image processing and report generator software (Nippon Avionics Co., LTD, Ikonobecho, Japan). The average environmental temperature was entered into the camera settings and it was a reference in each video as recommended in the literature [44]. From the videos recorded, frames with the best image quality were selected during the following phases of each session: (i) grooming; (ii) mounting; (iii) riding exercises; (iv) stationary exercises; (v) reward (see Table 1). A baseline image was recorded immediately before the starting of all experimental procedures in the same area as the sessions. All images were scanned from a distance of one metre. Temperatures of the lacrimal caruncle on both eyes of each horse were analysed with a method described by Redaelli et al., 2014 [45]. 

#### 2.6.4. Heart Rate and Heart Rate Variability

In order to record continuously the heart rate (HR) and heart rate variability (HRV) using the beat-to-beat interval (RR), horses were fitted with a girth enabling the attachment of a Polar Equine^®^ RS800 (Polar Electro Oy, Professorintie 5, 90440 Kempele, Finland). The girth was secured around the horse’s thorax to the left-hand side girth region and the electrodes were moistened with abundant transmission gel to improve the horse skin and coat conductance to maximize data recording accuracy. A heart rate monitor was fitted 5 min before the session started and removed 5 min after the end of the session. At the end of each session, the stored data were downloaded onto a computer through an infrared port (IrDa) for storage and later analysis. Data were analysed with Polar ProTrainer 5 software. The graphs were evaluated visually and corrected in case of too high or low peaks, or flat lines (plateau), considering a percentage of errors identified that did not exceed 6% as recommended in the literature [46]. HR and its oscillations (high-frequency band—HF, low-frequency band—LF, very low frequency band—VLF, LF/HF ratio), the standard deviation of RR interval (SDRR) and root mean square of successive RR differences (RMSSD) were calculated from the RR interval. For each phase of the sessions (see Table 1), these parameters were expressed as means and submitted to statistical analysis.

### 2.7. Statistical Analysis

Data were analysed using STATA 12.1 and SAS 9.4. We analysed hormonal (ACTH, cortisol, and catecholamines) differences between pre vs post-session using the Wilcoxon test for paired data and we investigated significant differences between ASD and TD sessions by sign test for paired data. Physiological (infrared thermography and heart rate and heart rate variability data), as well as behavioural parameters, were analysed using the Wilcoxon test for paired data. 

Multivariate analysis was performed using a linear mixed-effect model (LMM) using SAS 9.4 (PROC MIXED). This statistical model, containing both fixed effects and random effects, is very useful for repeated measurements on the same statistical units, as in our case [47]. Dependent variables included data from behavioural analysis, thermographic images, and heart rate monitor while explanatory variables included horse features (sex, age, experience), sessions (ASD vs. TD), and session phases. Horse age and experience were categorised considering median values (2–5 years vs. 6–14 years of experience in EAI). We included a random effect due to horse and the variance components were chosen as the best covariance structure. The normality of the residuals was checked using a normal probability plot. Post-hoc analysis was performed using adjusted *p*-value for multiple comparisons using Dunnett’s T3 method [48]. For the fixed effect, the denominator degrees of freedom are determined from a Satterthwaite approximation.

## 3. Results

### 3.1. ACTH, Cortisol, and Catecholamine

**Figure 2:**
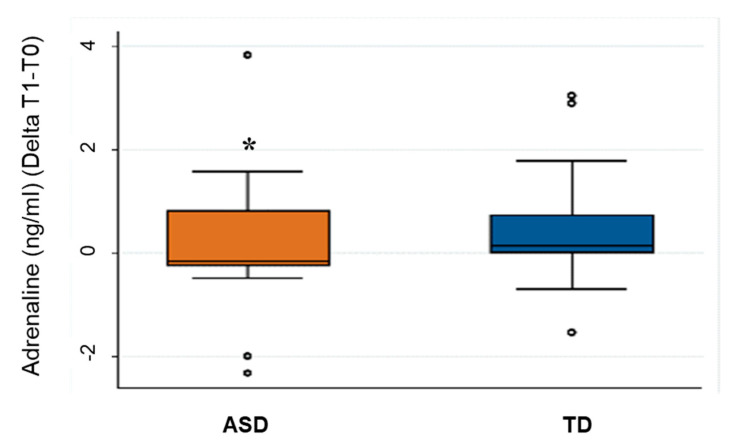
Differences (T1-T0) for adrenaline (ng/mL) in ASD and TD session. The figure shows the upper and lower quartiles (box), medians (horizontal line in the box), minimum and maximum values (whiskers), and outliers (individual points); * *p* < 0.05.

ACTH, cortisol, and catecholamines were within the physiological range in horses [49,50,51]. Plasma adrenaline, noradrenaline, dopamine, and ACTH responses to exercise were highly and positively correlated (rho_s_ > 0.7; *p* < 0.05) as described in the literature [52]. We considered the delta (T1-T0) and we used the Sign test to investigate differences between ASD and TD group. No differences were found in ACTH, cortisol, noradrenaline, and dopamine between the two groups (ASD vs. TD), while horses showed a significantly smaller increase of plasma adrenaline concentration in ASD sessions compared to TD sessions (*p*-value< 0.05) (Figure 2).

### 3.2. Behaviour Analysis

**Figure 3:**
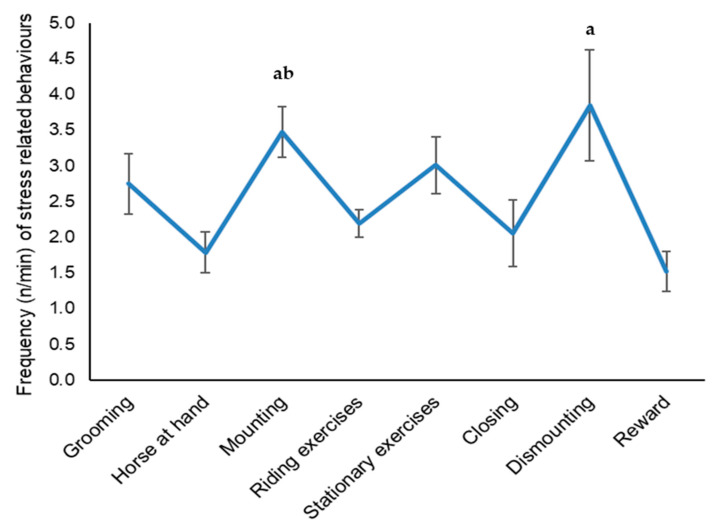
Frequency (n/min) of stress-related behaviours in horses in the different phases of the sessions (mean ± SEM). a: >“horse at hand, “closing”, and “reward” (all ps < 0.05); b: >“riding exercises” (*p* < 0.05).

Multivariate analysis on 19 horses using the LMM model showed no significant differences between sessions (ASD vs. TD), but only among different phases (see Type III Tests of Fixed Effects in Table 3, Figure 3). Post-hoc analysis pointed out a significant increase in stress-related behaviour frequency between mounting and dismounting phases compared to “horse at hand” (*p*-value = 0.0005 and 0.0052 respectively), “closing” (*p*-value = 0.0028 and 0.025), and “reward” (*p*-value = 0.0005 and 0.0053 respectively) and also between “mounting phase” compared to “riding exercises” (*p*-value = 0.0383). The normality of the residuals was confirmed using normal probability plots.

### 3.3. Eye Temperature

The LMM performed on the detected periocular temperatures, showed that there were no significant differences between sessions and phases. Residual analysis and their normality confirmed the validity of the applied mixed model. However, when we introduced the baseline eye temperature, collected before the starting of experimental procedures, ANOVA showed that there was a rise in eye temperature in the grooming phase compared to baseline in both ASD and TD sessions, but this difference became significant only in ASD sessions (*p*-value < 0.005).

### 3.4. Heart Rate and Heart Rate Variability (a Subset of Horses)

Only data from 11 horses were available for the final analysis because of interferences with the recording device. HR correlated with HRV inversely, as described in the literature [53], and HR had a physiological trend during sessions due to horse exercises with higher values during animal motor activities. The statistical analysis (LMM) pointed out significant differences among phases for HR (*p*-value < 0.001), LF/HF (*p*-value < 0.001) and RMSSD (*p*-value = 0.0002), but only RMSSD showed a significant difference between ASD and TD sessions (*p*-value = 0.0004) (Table 4). Moreover, an effect due to age and experience was found, with HR and RMSSD being significantly lower in older horses (≥18 years old) which were also those with more than 6 years of experience in EAI.

## 4. Discussion

Our study aimed to assess physiological and behavioural markers of stress commonly used in equitation science [54]. In particular, we aimed at integrating behavioural and physiological indices of stress during EAI sessions involving children with ASD to monitor horse welfare. Our results indicate that children with ASD do not impact the horse’s welfare compared with TD children of the same age, at least during a typical riding session like the one we set up.

We considered the main products of the hypothalamus-pituitary-adrenal (HPA) axis (plasma ACTH, and serum cortisol) and of the sympathetic–adrenal medulla axis (blood catecholamines), which are activated in response to stressful stimuli [55]. Many studies have previously investigated concentrations of these metabolites in horses exposed to both positive situations, such as sexual excitement or physical exercise [56,57], and negative ones (distress) such as restraint, isolation, or transportation [58] showing significant changes, depending upon the specific stimulation they were exposed to [52]. Even though the procedure we used was highly standardised and we limited seasonal and circadian rhythm fluctuations collecting blood samples during the same period of the year (November–January) and during the same time frame (14.00–16.00), our data showed a high degree of variability. This is expected, given the different riding centers, the different ages, and sexes of the horses involved [50], not to mention their life history. In any case, all parameters measured were well within the physiological range [59,60] in the two sessions and were not affected by the rider (ASD vs. TD session) suggesting that, on average, the riding session is not a stressful situation even when the rider is a child with ASD. These findings were confirmed by other authors: Johnson et al. investigated plasma ACTH, glucose, serum cortisol levels, and behaviour scores in horses involved in EAI sessions with military veterans affected by post-traumatic stress disorder (PTSD) and traumatic brain injury compared to sessions with experienced riders and they did not find any significant differences [61]. Similar results were described by Malinowsiki [62] and McKinney [63]: these researchers concluded that no significant differences were detected in delta salivary cortisol levels of horses comparing EAIs to traditional riding lessons or rest. 

In our study, we found a significantly smaller increase of adrenaline in ASD sessions compared to TD sessions. Adrenaline is responsible for the immediate response of the sympathetic branch of the ANS to stressors; therefore, this result suggests diminished arousal of the horse in EAI with children with ASD. This lack of arousal may be related to stimulus-evoked expectations resulting from environmental information that the horse receives from the handler, the child, and the overall setting [64]. These animals are employed in EAIs routinely, performing recurring tasks and activities. Therefore, the lack of activation of the sympathetic nervous system in ASD children may be due to a reduced expectation of novelty or an implicit solicitation to restrain their activation by the handler, as they are dealing with a child that needs special attention and care [65]. Lower activation of the HPA axis in horses has been suggested also by other researchers [66] investigating neuroendocrine responses during EAIs with children with physical disabilities, compared to recreational riding. In their study, endorphin and ACTH did not show significant changes, while cortisol was lower after EAI sessions. Our HR and HRV data support such hypothesis, providing an insight into the mental state of the animals involved in ASD and TD sessions. Analysing the main spectral components of the frequency domain, we noticed again a dominant vagal tone in ASD sessions compared to TD sessions. Indeed, we highlighted that the LF/HF ratio, which is a marker of sympathetic activity [67] was significantly reduced in the resting phases of the ASD sessions, compared to the TD sessions. Besides, RMMSD values, which represent vagal activity [46], were higher in ASD sessions, reaching a statistically significant difference compared to TD sessions. 

In our study, we also investigated eye temperature using infrared thermography-IRT. As this parameter is not influenced by exercise per se, it may be effective in evaluating the activation of the ANS, without being confounded by locomotor activity [68]. According to our results, the horse’s eye temperature did not show significant differences between ASD and TD sessions. We only highlighted a peak in the grooming phase compared to baseline, which became significant in ASD sessions, suggesting a physiological response of the animal as it started being groomed at the beginning of the EAI session. The handler gently restrained horses, while children groomed them. Horses were familiar with grooming procedures and they did not show any escape reaction.

It is hard to hypothesize the ultimate physiological significance in the increase of eye temperature, which is probably due to the horse’s awareness that the riding session is going to start: the grooming phase may act as a cue [69]. Interpretation of eye temperature changes in animals is still under debate. An increasing number of studies have found correlations between the rise of eye temperature and aversive stimuli and it appears to be indicative of alerting, anxiety, and possible discomfort [70,71]. Meanwhile, other researchers have highlighted that an increase in eye temperature may be related to positive emotional states [44]. In conclusion, IRT may be a useful tool to assess arousal although it fails to discriminate the positive vs the negative emotional content and behavioural cues may be needed to interpret these results [72].

When considering horse behaviours, no significant differences were found between ASD and TD sessions. However, an interesting result was found when analysing stress-related behaviours during the different phases of each session. In particular, a higher frequency of behaviours indicative of stress was found in the stationary phase, while resting phases, especially mounting and dismounting, appeared as the most challenging for the animal. It is possible to hypothesize that ill-fitted tack, type of handler restraint or children not very experienced with the horse may cause discomfort during mounting and dismounting. We conclude that commonly used behavioural requests in the different phases of the riding session should be managed with much more attention by the horse handler during EAI sessions to avoid stressful situations for the animal.

### Limitations of the Study

The current study was designed to investigate the effects of EAI sessions with children with ASD vs. TD children on the welfare of the horses involved, investigating both physiological and behavioural signals of stress commonly used in equitation science [15,34,35,36,37,38,39,40,41]. We acknowledge that the animal sample was very heterogeneous in terms of age, sex, and breeds of the horses enrolled in the study. This point necessarily affected the reliability of haematological and cardiac data [50]. Moreover, in equids, several confounding factors may influence the repeatability and reliability of HRV quantification [73]. Even the transmission of data from the Polar Equine RS800 was not always efficient and eight horses had an incomplete dataset. Environmental conditions affected the quality of IRT images because sessions were performed in outside paddocks. 

Lack of information about the long-term anamnesis of the horses involved, especially concerning the training methods used before their involvement in EAI practice and the nature of the previous experience they had in EAI, make a harder interpretation of the behavioural data. Horses enrolled in the study were 17 years old on average and it is well known that the use of aversive training methods was very common 10–15 years ago [68]. Moreover, they were assessed only twice, once with a child with ASD and once with a TD child: nowadays, no data are available about the variability of behavioural and physiological responses in horses to different children. Furthermore, we did not code child and handler behaviours performed during sessions including grooming, mounting, and dismounting phases to enhance the understanding of reactions in horses. Therefore, it cannot be ruled out that any difference between groups (ASD vs. TD) may be caused by a difference in IQ.

Currently, researchers have highlighted that lack of control over environmental factors may induce a psychological condition called learned helplessness [68]. The horse can show apathy and may only be compliant with the trainer’s requests. Some authors have suggested that learned helplessness may be related to depression-like conditions and anhedonia [39]. Thus, the absence of both conflict behaviour and physiological arousal is not necessarily indicative of a good welfare status [54]. The presence of positive emotions could be used as an important endpoint to assess animal’s welfare. Further studies are needed to investigate this aspect.

## 5. Conclusions

In conclusion, stress responses in horses involved in EAI’s were not affected negatively by short EAI sessions with ASD children compared to TD children of the same age and experience. Lack of novelty expectation during sessions with ASD children is likely to cause lower sympathetic tone, compared to sessions with TD children, an observation that requires to be studied further for the potential implications on horse’s welfare. Moreover, we noticed that some phases of the sessions seem to be more stressful for the horse (e.g., mounting and dismounting phase) requiring special attention by the animal handler. The results of this study appear important to refine methodologies in EAIs, encouraging professionals involved in animal-assisted interventions towards a greater awareness of horse’s welfare needs, which could enhance their role as emotional mediators.

Future large-scale studies need to be addressed to investigate positive emotion indicators in horses during EAI sessions and their reactions to human behaviours performed by different people.

## Figures and Tables

**Table 1:** Session description: phases and their duration and activities performed.

Phase	Duration (min)	Description of the Activity and Main Goals
Grooming	5’	Child’s approach to the horse and first contact; child’s knowledge of the horse (morphology, behaviour); main security rules; grooming techniques (brushing the body of the horse) and saddling
Horse at hand	5’	The child leads the horse with a lead rope around the arena
Mounting	-	The horse stops and the child mounts the horse
Riding exercises	10’	Learning riding basic elements (walk). Performing exercises while riding the horse (rotating/bending, outstretching upper arms and trunk)
Stationary exercises	5’	Performing exercises on the horse (horse halted). Games such as rods, cones, or balls are used
Closing	5’	Riding the horse around the arena
Dismounting	-	The horse stops and the child dismounts
Reward	-	The child rewards the horse (e.g., carrot, sugar, hay, etc.)

**Table 2:** Horse’s stress-related behaviours scored during the sessions.

Behaviour	Description
Head nodding	The horse repetitively moves its head vertically (>3 movements up and down)
Head shaking	The horse tosses its head in sudden bouts
Head tossing	Head lowered with the ears pinned back interrupted with momentary sharp tossing or rotating gestures of the head
Head raised/high	Head held higher than the normal carriage with nose extended upward and with a slight extension of the neck
Head down	The horse held its nose below its belly-line; neck may be stretched out with nose pushed forward
Ears pinned back	Ears pressed caudally against the head and neck
Snorting	Forceful expulsion of air through the nostril incidentally preceded by a raspy inhalation sound
Lip play	The horse moves its upper lip up and down without making contact with an object, or the horse smacks its lips together
Tongue play	The horse sticks out its tongue and twists it in the air
Chomping the bit	Any mouth or tongue manipulation of the bit independent of the rider’s use of the reins
Lip/Teeth rubbing	The horse rubs its upper lip or its upper teeth repetitively against the arena wall
Head bumping	The horse bumps or attempts to bump its head against the side walker or the instructor
Biting leads	The horse bites or attempts to bite the side walker/instructor the lead rope
Avoidance/Halt	The horse stops walking; cessation of movement of all feet, or backward movement
Pawing	The horse hits the ground with the paws
Tail swishing	Any exaggerated movement of the tail, usually more of a wringing motion than a rhythmic or directed swishing (no insect present)

**Table 3:** Results of the Type III Tests of Fixed Effects for stress-related behaviors.

Effect	DFnum	DFden	F	Pr > F
Phase	7	284	6.43	<0.001
Group (ASD vs. TD)	1	284	0.52	0.4733
Group*phase	7	284	1.61	0.1327
Age	1	18.8	0.38	0.5434
Experience (exp)	1	18.8	1.40	0.2509
Sex (M/F)	1	18.8	0.10	0.7592
Phase*age	7	284	2.07	0.0468
Phase*exp	7	284	2.00	0.0552
Phase*exp*M	8	96.8	2.06	0.0475
Phase*age*exp	8	96.8	2.06	0.0475

**Table 4:** Results of the Type III Tests of Fixed Effects for HR, LF/HF, RMSSD.

Effect	HR		LF/HF		RMMSD	
	F	Pr > F	F	Pr > F	F	Pr > F
Phase	48.29	<0.0001	6.76	<0.0001	4.53	0.0002
Group (ASD vs. TD)	1.52	0.2201	1.49	0.2243	13.42	0.0004
Group*Phase	3.85	0.0008	0.56	0.7909	1.01	0.4297
Age	2.76	0.1298	0.37	0.5566	7.91	0.0187
Phase*age	4.04	0.0005	1.39	0.2149	6.38	<0.0001
Group*age	3.48	0.0643	1.86	0.1751	0.27	0.6058
Group*phase*age	0.93	0.4836	0.34	0.9348	1.02	0.4177
Age*exp	0.07	0.7997	0.01	0.9275	1.17	0.3055
Phase*age*exp	2.58	0.0163	1.23	0.2894	0.71	0.6658
Group*age*exp	0.10	0.7539	0.61	0.4370	0.02	0.8764
Group*phase*age*exp	2.62	0.0147	0.56	0.7878	1.04	0.4053

## Data Availability

All relevant data is listed in the manuscript. Additional information can be requested from the authors upon reasonable request.
